# Gut Microbial Composition Is Associated with Symptom Self-Report in Trauma-Exposed Iraq and Afghanistan Veterans

**DOI:** 10.1089/neur.2024.0011

**Published:** 2025-01-08

**Authors:** Y. Irina Li, Kathleen Pagulayan, Holly Rau, Rebecca Hendrickson, Abigail G. Schindler

**Affiliations:** ^1^ Northwest Mental Illness Research, Education and Clinical Center, Veterans Affairs (VA) Puget Sound Health Care System, Seattle, Washington, USA.; ^2^ Department of Anesthesiology, VA Puget Sound Health Care System, Seattle, Washington, USA.; ^3^ Department of Rehabilitation Medicine, University of Washington, Seattle, Washington, USA.; ^4^ Department of Psychiatry and Behavioral Sciences, University of Washington, Seattle, Washington, USA.; ^5^ Graduate Program in Neuroscience, University of Washington, Seattle, Washington, USA.; ^6^ VA Northwest Geriatric Research Education and Clinical Center, VA Puget Sound Health Care System, Seattle, Washington, USA.; ^7^ Department of Medicine, University of Washington, Seattle, Washington, USA.

**Keywords:** gut microbiome, military deployment, polytrauma clinical triad, trauma, Veterans

## Abstract

Iraq and Afghanistan War-era Veterans are at elevated risk for physical injuries and psychiatric illnesses, in particular the polytrauma triad of mild traumatic brain injury (mTBI), post-traumatic stress disorder (PTSD), and chronic pain. The gut microbiome has been implicated in modulation of critical processes beyond digestion, including immune system functioning and stress responsivity, and may be an important factor in understanding physical and mental health outcomes following deployment and trauma exposure. However, minimal research to date has sought to characterize gut microbiome composition in this population. Male Veterans of the conflicts in Iraq and Afghanistan who previously completed a Veterans Affairs’ comprehensive TBI evaluation were enrolled in the current study. Participants completed self-report measures of PTSD symptom severity, pain intensity and interference, fatigue, cognitive symptoms, substance use, and sleep quality. They also submitted fecal samples, and metagenomic sequencing was used to calculate alpha and beta diversity and taxonomic microbial composition. Associations between microbiome data and clinical variables were then examined. Alpha and beta diversity measures were not significantly correlated with clinical outcomes. Fatigue, post-concussive symptoms, executive function symptoms, and cannabis use were associated with differences in gut microbial composition, specifically Verrucomicrobiota. Together, results suggest that altered gut microbiome composition is associated with psychiatric and cognitive symptoms in Veterans and highlight a potential new therapeutic target of interest. Future research is needed to examine whether probiotic treatment is effective for reducing symptoms common in this clinical population.

## Background

The negative impact on physical and mental health following deployment to war zones among U.S. military personnel and Veterans has been well-documented in the empirical literature.^1–4^ Data from the Millennium Cohort Study—a prospective longitudinal study by the Department of Veterans Affairs (VA) and Department of Defense—showed that deployment is associated with increased cardiovascular risk, smoking initiation, chronic pain, and greater medical illness burden.^5–9^ Likewise, in a large-scale review, Pietrzak et al. found increased rates of post-traumatic stress disorder (PTSD), depression, and anxiety post-deployment.^10^ Despite greater understanding in recent years of the negative impact of deployment and combat exposure on health outcomes, the full breadth of underlying mechanisms is still being uncovered, and effective treatment options are limited. More recently, the role of gut microbiome in the modulation of health and disease processes has garnered increasing attention.^11^ As mounting literature delineates complex interactions between microbiota and human hosts in contributing to disease processes, there is a need for better characterization of gut microbiome composition in Veterans to enhance our understanding of its possible relationship to post-deployment symptoms and point to novel intervention targets.

Service members involved in the conflicts in Iraq and Afghanistan experience greatly elevated rates of co-occurring physical and mental health conditions,^1,12^ with mild traumatic brain injury (mTBI), PTSD, and pain co-occurring at such high rates in this Veteran population that it is commonly referred to as the “polytrauma clinical triad.”^13^ Increasing evidence indicates that the presence of some combination of the polytrauma triad makes individuals particularly vulnerable to additional medical and psychiatric comorbidities and leads to greater overall chronic disease burden.^14–16^ For example, the combination of deployment-related mTBI and PTSD is associated with greater PTSD and depressive symptoms, pain interference, and poorer sleep quality.^17^ Newly emerging evidence also suggests that mTBI sustained in the context of combat leads to poorer outcomes and greater disability relative to those service members who did not sustain a TBI, even a decade later.^18^ Thus, greater understanding of multifactorial influences on long-term outcomes in this unique and complex population is critical for the development of effective interventions to better improve outcomes.

The gastrointestinal (GI) system may be particularly vulnerable to the effects of blast exposures, as well other deployment-related factors.^19^ Indeed, there is growing recognition that deployment-related factors can impact GI health.^20,21^ Critically, an accumulating body of literature suggests that a positive link exists between GI distress and mental health disorders,^22,23^ which may, in part, contribute to the complex clinical picture of overlapping adverse physical and psychological outcomes in returning service members and Veterans.

The association between psychological and GI distress has led to increasing attention around the role of the microbiome in the modulation of physical and mental health symptoms. The human microbiota, referring to all microorganisms in and on the human body, is made up of bacteria, archaea, fungi, protozoa, and bacteriophages.^24^ The majority of bacteria that make up the human microbiome reside in the gut and can be impacted by a wide range of factors, including genetics, environment, early childhood experiences, physical illness, physical activities, and medications.^25–28^ In turn, the gut microbiome plays a crucial role in regulating numerous functions important for health and survival, including digestion, maintaining the intestinal epithelium, resistance to pathogens, and immune system functioning.^29^ In addition, the gut microbiome is thought to play an important role in bidirectional communication between the gut and the brain (the microbiota–gut–brain axis [MGBA]), particularly in the context of physical and psychological trauma.^30–32^ Bidirectional communication takes place between the gut microbiome, GI tract, and central nervous system and includes neuroendocrine and neuroimmune systems, sympathetic and parasympathetic arms of the autonomic nervous system (including the enteric nervous system), and the vagus nerve. Gut microbiota interact with, metabolize, and/or produce a variety of biochemical compounds, including cytokines, neurotransmitters, neuropeptides, endocrine messengers, and microbial metabolites such as short-chain fatty acids.

While the literature is still emerging, studies suggest that the gut microbiome is implicated in regulation of affective processes such as anxiety and depression-related symptoms. Modification of the gut microbiome via colonization of donor gut bacteria from mice with enhanced anxiety phenotype has been shown to elicit anxiety-like behaviors in non-anxious mice.^33^ Alteration of the gut microbiome via administration of beneficial bacteria such as *Lactobacillus rhamnosus* or *Bifidobacterium longum* has led to decreased anxiety and depressive behaviors in rodent models.^34,35^ In humans, probiotic administration of *Lactobacillus* or *Bifidobacterium* species has been shown to reduce anxiety and depression symptoms,^35^ supporting MGBA as a potentially important modulatory link between physical and mental health.

Stress exposure may impact gut microbial composition via the release of stress hormones or increased gut permeability, altering the microbiota habitat.^36,37^ Gut microbiome composition can be influenced by both distal stressors, such as early life experiences, and proximal stress, such as blast-exposure and TBI.^36–38^ More specifically, gut microbiome has been shown to play a role in the modulation of systems involved in affective signaling, including the hypothalamic–pituitary–adrenal axis,^39^ which may increase susceptibility to emotion dysregulation and subsequent risk for the development of psychiatric conditions, including PTSD,^40^ following exposure to trauma.^41^ Importantly, in Veteran populations, there is evidence linking exposure to traumatic stressors (i.e., military deployment) and mental health conditions to altered gut microbiota composition.^42^ Additional research is needed to better understand and characterize the nature of these associations.

In animal models, TBI has been shown to induce changes in microbiome composition including decreased diversity and abundance of *Lactobacillus gasseri, Ruminococcus flavefaciens*, and *Eubacterium* and increased abundance of *Eubacterium sulci* and *Marvinbryantia formatexigens*.^37,43^ In humans, GI dysfunction and complaints following TBI are well-documented,^44^ yet little research has directly examined the relationship between TBI and microbiome composition. In patients with moderate-to-severe TBI, one study found no association between microbiota diversity in a sample of Veterans^45^; a separate study, however, found reduced *Prevotella* and *Bacteroides* species and increased *Ruminococcaceae* in patients residing in permanent care facilities.^46^ To our knowledge, and according to a recent 2022 literature review,^47^ no studies have examined microbiome changes following mTBI in humans.^39^

Taken together, evidence suggests that the gut microbiome may be an important factor in understanding the trajectory of physical and mental health outcomes of military personnel following deployment, particularly in the context of the polytrauma clinical triad. The limited available literature on gut microbiome in Veterans has predominantly focused on broad characterization,^42,48–50^ and has not focused on psychological or mental health outcomes nor specifically on combat Veterans of the Iraq/Afghanistan conflicts. Therefore, enhancing our understanding of gut microbiota composition in this unique population is critical in elucidating potential pathogenic processes involved in the polytrauma clinical triad and potentially informing future therapeutic targets. The goals of the present study were to examine (a) the gut microbiome profile in a sample of combat deployed Iraq/Afghanistan Veterans and (b) potential associations between gut microbial composition and physical and mental health symptoms.

## Methods

### Study procedures

Prospective study participants were identified via a review of the VA Computerized Medical Record System for Veterans who completed a Comprehensive TBI Evaluation (CTBIE) at a large metropolitan Veterans Health Administration facility between 2019 and 2021. Individuals were eligible for the study if they were an OEF/OIF/OND Veteran between the ages of 18 and 65 with a remote history of a blast-related mTBI or no history of TBI in adulthood and were able to read, speak, and comprehend English. As this was an exploratory study with a relatively small sample, only male Veterans were eligible given that the majority of Iraq/Afghanistan Veterans were male. Exclusion criteria were (a) acute mTBI (i.e., injury within the past 3 months), (b) moderate or severe TBI diagnosis, (c) history of serious mental illness (e.g., schizophrenia, schizoaffective disorder, or bipolar disorder), (d) current (defined as past 3 months) substance use disorder diagnosis, (e) current neurological disorder that could impact cognitive functioning, (f) high risk of suicide, or (g) failure of two or more items on a brief 6-item cognitive screener. The study was approved by the local institutional review board.

After providing informed consent, study participants were asked to complete an online survey and provide a fecal sample collected at home using the OMNIgene GUT self-collection kit. Fecal samples were returned to the research team via overnight mail delivery and then sent to Diversigen for sample processing and bioinformatic analysis. Online surveys were completed within 3 days of sample collection included questions about demographic information, medical and psychiatric history, and current use of medications and over-the-counter supplements.

### Clinical symptom measures

TBI history was gathered from Veterans’ electronic health records via the CTBIE, a VA-mandated semi-structured interview that is completed by a physician as part of clinical care. PTSD symptom severity was assessed with the PTSD Checklist for DSM-5.^51^ Depression symptom severity was assessed with the Patient Health Questionnaire-9.^52^ Fatigue was measured with the Patient-Reported Outcomes Measurement Information System (PROMIS)^53^ Fatigue Short Form 6a. Pain severity was measured using the Pain Intensity Short Form 3a^54^ (while interference of pain on daily activities was assessed with PROMIS-Pain Interference Short Form 8a^55^). The Neurobehavioral Symptom Inventory^56^ was used to assess current (past two weeks) post-concussive symptoms. Current cognitive symptoms were assessed with the Traumatic Brain Injury-Quality of Life Measurement System (TBI-QOL) Attention and Concentration, Executive Functioning, and Learning and Memory scales.^57,58^ Sleep quality was assessed with the Pittsburgh Sleep Quality Index.^59^ Current substance use was assessed with the Alcohol Use Identification Test,^60^ Cannabis Use Disorder Identification Test-Revised,^61^ and Drug Abuse Screening Test.^62^ For all participant-reported symptom measures with exception of the TBI-QOL, higher scores reflect greater symptom severity.

### Fecal sample collection

The OMNIgene GUT self-collection kit was used by study participants at home to collect stool specimen. This system allows for rapid homogenization and stabilization of microbial DNA at point-of-collection, eliminating cold chain requirement (sample storage at ambient room temperature for up to 60 days). Briefly, each participant was instructed to (1) deposit stool into a collection bowl suspended over a toilet, (2) transfer an aliquot of stool using the provided scoop into the collection tube (contains homogenization bead and solution to stabilize microbial DNA), (3) shake the sealed tube for 30 sec, and (4) return sample in pre-addressed mailing envelope to the VA study site.

### Fecal sample processing

Fecal samples were mailed in bulk to Diversigen for DNA extraction and metagenomic analysis using their BoosterShot Shallow Shotgun Sequencing. In brief, DNA sequences were aligned to a curated database containing all representative genomes in RefSeq for bacteria. Alignments were made at 97% identity against all reference genomes. Every input sequence was compared with every reference sequence in the Diversigen Venti database using fully gapped alignment with BURST. Ties were broken by minimizing the overall number of unique operational taxonomic units (OTUs). For taxonomy assignment, each input sequence was assigned the lowest common ancestor that was consistent across at least 80% of all reference sequences tied for best hit. Samples with fewer than 10,000 sequences were discarded (no samples in the current study were discarded). OTUs accounting for less than one millionth of all strain-level markers and those with less than 0.01% of their unique genome regions covered (and <0.1% of the whole genome) at the species level were discarded. The number of counts for each OTU was normalized to genome length.

### Data analysis

Centered log ratio transformations were conducted on phylum-level compositional data to account for non-normality in distribution of microbiome data. We then conducted nonparametric Spearman correlations of gut microbiome parameters with clinical outcome variables. An additional analysis examined rank-biserial correlations between gut microbiome parameters and self-report medication use. We elected to specifically examine medications with strong documented effects on the gut microbiome, which in our sample were medications with serotonergic action (selective serotonin reuptake inhibitors, selective serotonin receptor agonists, serotonin and norepinephrine reuptake inhibitors, tricyclic antidepressants), angiotensin-converting enzyme (ACE) inhibitors, alpha and beta blockers, and vitamin D.^63,64^ Self-reported health conditions were categorized into mental health conditions (depression, PTSD, generalized anxiety disorder, and ADHD), neurological disorders (migraine/headaches, stroke or aneurysm, brain tumor, brain surgery, epilepsy or seizures, multiple sclerosis, Parkinson’s disease, and other neurological illness), GI disorders (gastroesophageal reflux disease [GERD], gluten intolerance, celiac disease, lactose intolerance, IBS [irritable bowel syndrome], IBD [inflammatory bowel disease], and other GI problems), endocrine disorder (liver disease, kidney disease, hepatitis C, thyroid disease, low testosterone, growth hormone deficiency, and other endocrine condition), sleep disorders (insomnia and sleep apnea), and other health conditions (hypertension, hypercholesterolemia, hyperlipidemia, cardiovascular disease, heart attack, diabetes, cancer, asthma, chronic obstructive pulmonary disease, B12 deficiency, musculoskeletal pain, arthritis, gout, and lupus). Correlational analyses were conducted using SPSS 26.0.0 with correction for multiple comparisons using the Benjamini–Hochberg false discovery rate method.

Alpha diversity indices assessed were observed species richness, Chao1, and Shannon, measuring diversity within samples. Beta diversity (measuring dissimilarity between samples) was evaluated using Bray Curtis dissimilarity matrix and visualized using principal coordinate analysis (PCoA). Biplots were used to visualize associations between participant samples and clinical self-report variables. Biplot analysis was conducted in R (2022.07.1).

## Results

### Participant demographics and polytrauma clinical triad assignment

**FIG. 1.**
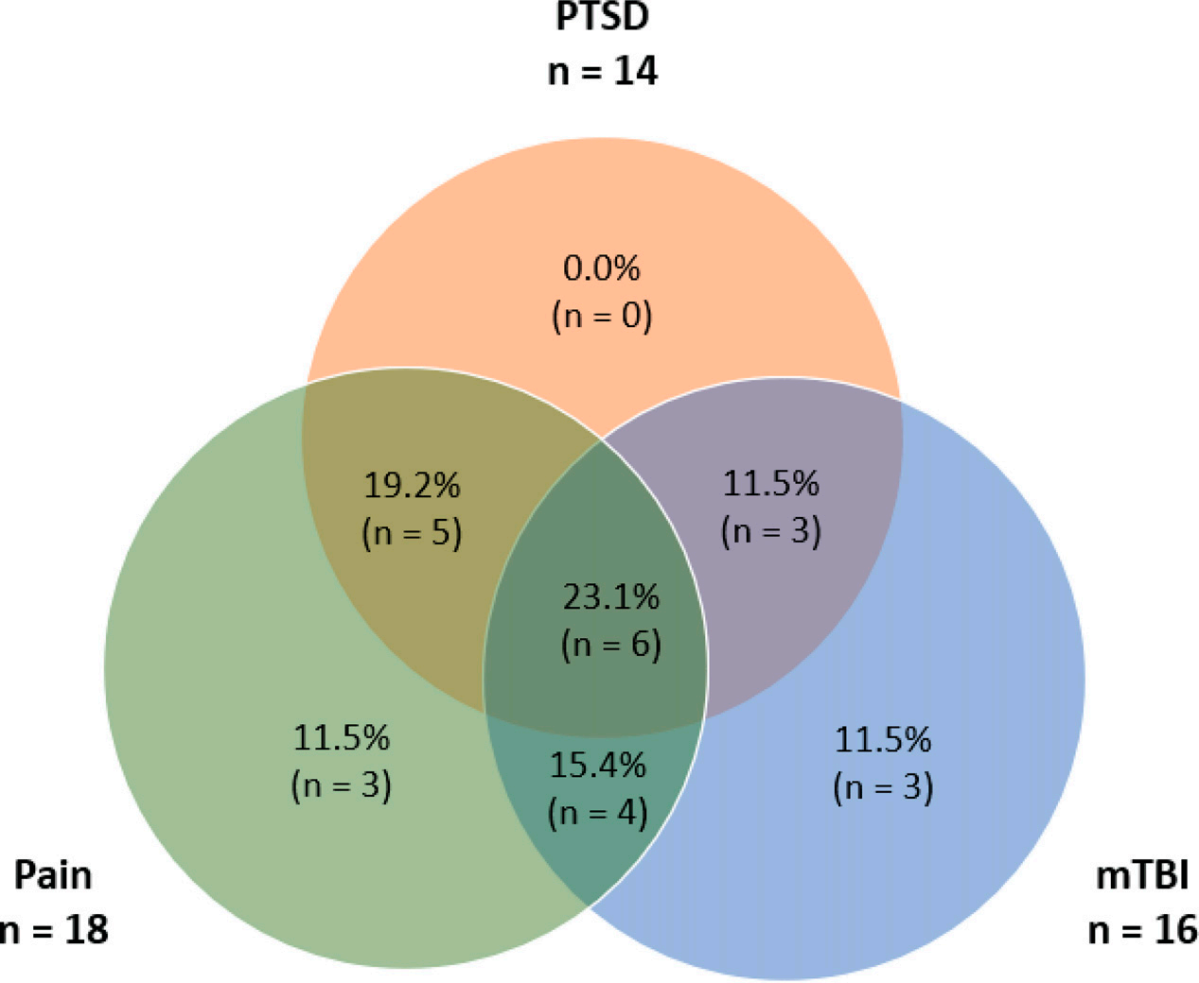
The polytrauma clinical triad: Distribution of study participants with clinically significant post-traumatic stress disorder (PTSD) symptoms, chronic pain interference, and mild traumatic brain injury (mTBI).

A total of 26 participants (mean age = 41.9 years [range 30–55], 50% White, 12% Black or African American, 4% Asian or Asian American, 4% American Indian or Alaskan Native, 8% Native Hawaiian or Pacific Islander, 12% Mixed, and 12% Other; 15% Hispanic ethnicity) were enrolled in the study. Table 1 summarizes demographic data and clinical symptom measures of study participants. Approximately half (57%) of participants had three or more deployments and spent in total one or more years on deployment. Figure 1 shows the proportion participants with polytrauma clinical triad symptoms in this sample. Approximately 62% of Veterans had a history of at least one mTBI during deployment. Approximately 81% of the participants had a self-reported lifetime diagnosis of PTSD, and ∼54% met the clinical cutoff for probable PTSD diagnosis on the PTSD Checklist for DSM-5 at the time of the study (i.e., based on a clinical cutoff of 33^65^). Clinically significant pain intensity was reported by 69% of participants, and 73% reported clinically significant pain interference (i.e., based on T-scores higher than one standard deviation above community norms).^66^ Only two participants did not endorse clinically significant PTSD symptoms, pain symptoms, or history of mTBI. One or more GI disorder diagnosis was reported by 42% of Veterans (GERD: 6/26; IBS: 5/26; IBD: 1/26).

**Table 1.** Characteristics of Study Participants (*N* = 26)

Demographic variables	
Age (M [SD])	41.9 (7.2)
Race (*n*)	
White	13
Black	3
Asian	1
American Indian or Alaskan Native	1
Native Hawaiian or Pacific Islander	2
Mixed race	3
Other	3
Hispanic ethnicity (*n*)	4
Education level (*n*)	
High school graduate or GED	4
Some college	6
Associate’s degree	3
Bachelor’s degree	7
Graduate degree	6
Medications with influences on the gut microbiome (*n*)	
Serotonergic system acting	10
ACE inhibitors	5
Alpha or beta blockers	7
Vitamin D	12
*Clinical variables*	*M (SD*)
Number of self-reported lifetime health conditions	
Mental health disorders	2.2 (1.3)
Neurological disorders	0.8 (0.6)
Gastrointestinal disorders	0.6 (0.9)
Endocrine disorders	0.2 (0.4)
Sleep disorders	1.0 (0.8)
Other health disorders	2.4 (1.4)
PROMIS	
Pain intensity	25.3 (8.6)
Pain interference	9.0 (2.0)
Fatigue	19.8 (6.3)
PSQI	11.8 (3.7)
PHQ-9	12.5 (7.5)
PCL-5	35.3 (19.5)
NSI	33.3 (17.0)
TBI-QOL	
Attention/concentration	16.9 (6.4)
Executive functioning	34.0 (10.2)
Learning/memory	18.6 (5.9)
AUDIT	3.5 (6.1)
CUDIT-R	2.4 (3.4)
DAST	1.1 (1.5)

Mental health disorders include depression, PTSD, generalized anxiety disorder, and ADHD. Neurological disorders include migraine/headaches, stroke or aneurysm, brain tumor, brain surgery, epilepsy or seizures, multiple sclerosis, Parkinson’s disease, and other neurological illness. Gastrointestinal disorders include GERD, gluten intolerance, celiac disease, lactose intolerance, IBS, IBD, and other gastrointestinal problem. Endocrine disorders include liver disease, kidney disease, hepatitis C, thyroid disease, low testosterone, growth hormone deficiency, and other endocrine condition. Sleep disorders include insomnia and sleep apnea. Other health disorders include hypertension, hypercholesterolemia, hyperlipidemia, cardiovascular disease, heart attack, heart surgery, diabetes, cancer, asthma, chronic obstructive pulmonary disease, B12 deficiency, musculoskeletal pain, arthritis, gout, and lupus.

ACE, angiotensin-converting enzyme; AUDIT, Alcohol Use Disorders Identification Test; CUDIT-R, Cannabis Use Disorders Identifications Test-Revised; DAST, Drug Abuse Screening Test; NSI, Neurobehavioral Symptoms Inventory; PCL-5, PTSD Checklist for DSM-5; PHQ-9, Patient Health Questionnaire-9; PROMIS, Patient-Reported Outcomes Measurement Information System; PSQI, Pittsburgh Sleep Quality Index; TBI-QOL, Traumatic Brain Injury-Quality of Life.

### Correlation between gut microbiome and self-reported clinical symptoms

Table 2 shows a heat map of the correlations of gut microbiome diversity and abundance parameters and polytrauma triad-related clinical outcome variables. Alpha diversity represents the within-sample diversity, observed species represent the microbiota richness, Shannon diversity takes into account both evenness and richness, and Chao1 diversity represents the microbiota richness abundance. Conversely, beta diversity captures the diversity between samples. In the analysis of alpha diversity measures, none were significantly correlated with any current clinical symptoms. While observed species number and Chao1 index were marginally negatively correlated with pain interference, fatigue, and sleep quality, results were not significant when adjusting for multiple comparisons. In the analysis of beta diversity measures, when correcting for multiple comparisons, no significant correlations were found (fatigue, sleep quality, and cannabis use were all marginally correlated prior to multiple comparison correction).

**Table 2.** Heat Map of Nonparametric Spearman Correlations Between Gut Microbiome Diversity Metrics and Clinical Outcome Measures

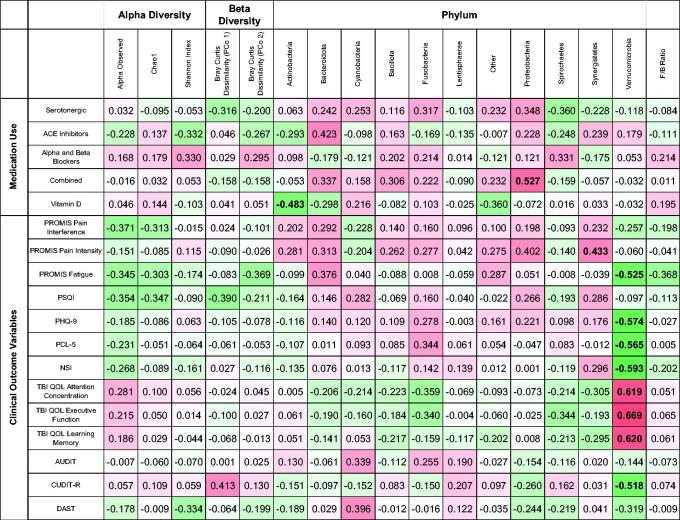

TBI-QOL measures are reverse scored.

Bold *r* values are significant at FDR 0.05.

At the phylum taxonomic level, abundance of Actinobacteria was significantly negatively correlated with use of vitamin D, and Proteobacteria was significantly positively associated with combined medication use. Verrucomicrobiota showed the strongest associations with self-reported symptoms, with decreased abundance associated with greater fatigue, depressive symptoms, PTSD symptom severity, post-concussive symptoms, cannabis use, as well as difficulties with attention, executive function, and learning and memory.

### Biplot analysis of gut microbiome diversity

**FIG. 2.**
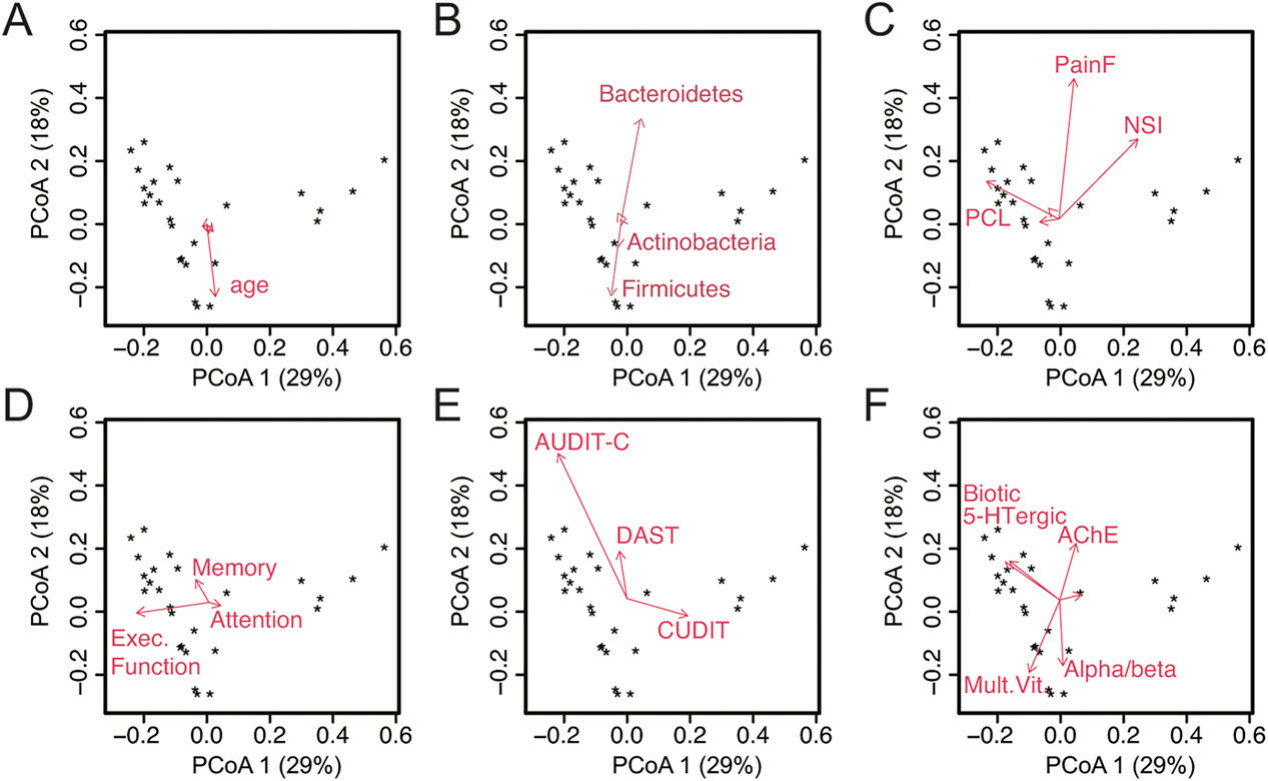
PCoA biplots displaying a dimensionality reduction of samples (e.g., PCoA generated from OTUs using Bray Curtis) and variables of interest in self-report measures with respect to the same set of coordinates. Variables of interest are projected as vectors with length of vector indicating strength of association and direction indicating degree of difference between PCoA axes. **(A)** Demographics. **(B)** Phylum-level taxonomy. **(C)** Polytrauma clinical triad self-report metrics. **(D)** Cognitive self-report. **(E)** Substance use self-report. **(F)** Medication class use self-report. LM, TBI-QOL learning and memory subscale; PCoA, principal coordinate analysis.

PCoA biplots were performed to further visualize and identify associations between participant fecal samples in relation to self-report measures (Fig. 2A–F). A biplot displays a dimensionality reduction of samples (e.g., PCoA generated from OTUs of microbiota using Bray Curtis) and variables of interest in self-report measures with respect to the same set of coordinates. Variables of interest are projected as vectors with length of vector indicating strength of association and direction indicating degree of difference between PCoA axes. Biplots are shown for (1) demographics (age, race, deployment length, deployment number, (2) phylum-level taxonomy, (3) polytrauma clinical triad self-report, (4) cognitive self-report, (5) substance use self-report, and (6) medication class use self-report (note, only variables with appreciable loading are included on the biplots). In regard to demographic variables, only age showed significant loading. Among phylum-level taxonomy, Bacteroidetes and Firmicutes were the strongest drivers of differences in microbiome composition at the phylum level and suggest that additional information may be gained by examining differences in composition on a more refined level such as genus or species (but due to limited sample number is outside the scope of the current study). Among clinical outcome variables, fatigue and PCS symptoms were most strongly correlated with microbiome composition. Of the cognitive self-report measures, executive functioning symptoms were the most strongly associated with differences in microbiome composition. Alcohol use, ACE inhibitor, alpha and beta blockers, and serotonergic medications were also associated with differences in gut microbiome composition.

## Discussion

This exploratory study sought to characterize gut microbiome composition in a sample of OEF/OIF/OND Veterans who present with high rates of mTBI, PTSD, and pain. We did not find significant associations between alpha and beta diversity measures and clinical outcome measures. These results are consistent with those reported by Brenner et al.,^45^ who found that when compared with Veterans with no or mild TBI, those with moderate or severe TBI did not differ in alpha and beta diversity measures, or taxonomic differences. Findings on alterations in gut microbiome following TBI have been mixed, with some studies suggesting a time-dependent course of change, with decreases in gut microbiome diversity immediately post-injury followed by a return to the pre-injury state.^45,67,68^ However, other studies have found evidence for more persistent changes in the gut microbiome.^46,69^ The present study suggests phylum-level differences in gut microbiome composition are present in trauma-exposed Veterans with a history of combat deployment. Deployment is associated with a number of environmental and psychiatric stressors, which may exacerbate or prolong changes in the gut microbiome following acute stress. There is also the possibility that even acute changes in the microbiome may contribute to pathogenic pathways such as neuroinflammation, intestinal permeability, and systemic inflammation,^70,71^ with further downstream consequences.

While the analyses did not reveal significant associations between measures of alpha or beta diversity and self-reported mental health or cognitive symptoms, a decreased abundance of the bacterial phylum Verrucomicrobia was strongly correlated with greater severity of PTSD and depressive symptoms, fatigue, and post-concussive symptoms. Verrucomicrobia has been positively linked with improved insulin sensitivity and glucose tolerance and has been proposed to be protective for metabolic health.^72,73^ Furthermore, our results are in line with a previous study on Veterans demonstrating a negative association between Verrucomicrobia abundance and PTSD self-report.^40^ Likewise, in a rodent model of PTSD, Verrucomicrobia exhibited both immediate and prolonged alteration in relative abundance following stress exposure.^74^ Together, our results are consistent with previous reports and raise the possibility that the prolonged stress associated with deployment (and repeated deployment for the majority of our sample), as well as chronic PTSD, may be associated with alterations in Verrucomicrobia abundance. Our findings provide preliminary support for altered gut microbiome composition in Veterans with a high rate of polytrauma clinical triad symptoms and highlight a potential new therapeutic target in this clinical cohort.

Our results further suggest that fatigue may also be linked with differences in gut microbial community composition. Persistent fatigue is a frequent complaint in conditions such as chronic pain and IBD and is a complex multifactorial clinical problem with a number of proposed pathways including central nervous system dysfunction, inflammation, dysregulation of sleep–wake mechanisms, and altered motivational and reward-processing functioning.^75,76^ Interestingly, bacteriotherapy via transcolonoscopic infusion of nonpathogenic enteric bacteria has been shown to improve symptoms in patients with chronic fatigue syndrome,^77^ suggesting that the relationship between fatigue and gut microbiome may be bidirectional. Fatigue is also commonly reported in Veterans with mTBI, particularly in the presence of comorbid sleep disturbances, depression, and chronic pain.^78,79^ Furthermore, insomnia is the most commonly reported symptom among Iraq/Afghanistan Veterans with PTSD,^80^ which contributes to high levels of daytime fatigue.^80^ In the present study, both fatigue and Bacteroidetes were aligned with fecal samples with similar gut microbiome characteristics, suggesting that examination of potential associations between fatigue and Bacteroidetes may be important for understanding clinical outcomes in the polytrauma clinical triad population.

Post-concussive symptoms were also a factor in accounting for similarities in microbial community structure in this sample. Patients with TBI are at greater risk for GI dysfunction and distress^81^; it has been suggested that downstream negative consequences of TBI, such as increased incidence of disease and disability, are rooted in part in dysregulation of the gut–brain axis following brain injury.^82,83^ Repetitive mTBI has been found to lead to changes in microbial composition in the rat jejunum, with mTBI rats exhibiting reductions in gut microbial diversity compared with controls.^69^ It is important to note that the findings have been somewhat inconsistent, with another study examining a model of repetitive mTBI in male mice demonstrating only small and transient alterations in the gut microbiome.^84^ A systematic review of animal and human studies suggests that TBI leads to alterations in gut microbiome composition, although the authors noted limitations involving small number of studies and sample sizes.^85^ While studies of uncomplicated mTBI in civilian populations have generally found that the vast majority of individuals recover quickly and with a relatively short-term course of post-concussive symptoms,^86,87^ more recent literature has suggested there is significant heterogeneity in outcome trajectory following mTBI even in civilian populations, with a subgroup of individuals continuing to experience symptoms and associated functional impairment one year post-injury.^88^ Together, this suggests that changes in gut microbiota composition resulting from neurotrauma could, in turn, contribute to further pathogenic processes such as dysregulation of immune functioning.^89,90^

### Limitations

This study has several important limitations. First, this was an exploratory study with a small sample size, which reduced our power to detect significant effects. In addition, this Veteran sample had high rates of comorbid mTBI, PTSD, and pain, consistent with other studies in this population. While this was expected given that our aim was to broadly characterize the gut microbiome in this population, high symptom burden and use of medications that may affect the microbiome preclude drawing conclusions about directionality of effects. There was also variability in the clinical characteristics in our sample, including in time since mTBI, PTSD symptom severity, and use of non-medication supplements, all of which are likely associated with differences in gut microbiota composition. In addition, it was beyond the scope of this study to assess other potentially important factors, including time since PTSD symptom onset and characteristics of military service (e.g., length/number of deployment[s]). Lastly, relative abundance of gut microbiota also demonstrates natural oscillations linked to circadian rhythmicity and the light–dark cycle.^90^ Variability in timing of fecal sample collection may make disentangling the effects of the light–dark cycle from observable differences in gut microbiota composition difficult.

## Conclusion

The limited available literature examining the relationship between the gut microbiome and physical and psychological outcomes in Veterans is mixed and requires further investigation. To our knowledge, this is the first study to examine the gut microbiome in a sample of Iraq/Afghanistan Veterans with high rates of polytrauma triad symptoms. Our results demonstrate potential alterations in gut microbiota composition associated with increased psychiatric and cognitive symptoms in Veterans following deployment to combat zones. These Veterans faced increased rates of deployment-related stress exposures as well as alterations in environmental factors such as living conditions, sleep patterns, dietary intake and eating patterns, and local microbiomes, which may place them at unique risk for changes in gut microbiome composition and downstream pathogenic processes. The present study joins an expanding body of literature examining the relationship between gut microbiota and physical and mental health in Veterans. Additional research is warranted to determine whether this pattern is specific to male combat Veterans and whether treatments targeting the gut (e.g., probiotics) have a salutary effect on symptoms common to this clinical population.

## Abbreviations Used

ADHD: attention deficit hyperactivity disorder
OEF: Operation Enduring Freedom
OIF: Operation Iraqi Freedom
OND: Operation New Dawn
PCS: Post-concussive symptoms
